# Perioperative, short-, and long-term mortality related to fixation in primary total hip arthroplasty: a study on 79,557 patients in the ­Norwegian Arthroplasty Register

**DOI:** 10.1080/17453674.2019.1701312

**Published:** 2019-12-13

**Authors:** Håvard Dale, Sjur Børsheim, Torbjørn Berge Kristensen, Anne Marie Fenstad, Jan-Erik Gjertsen, Geir Hallan, Stein Atle Lie, Ove Furnes

**Affiliations:** a The Norwegian Arthroplasty Register, Department of Orthopaedic Surgery, Haukeland University Hospital, Bergen;; b Department Clinical Medicine, University of Bergen, Bergen;; c Department of Surgery, Voss Hospital, Voss;; d Department of Clinical Dentistry, University of Bergen, Bergen, Norway

## Abstract

Background and purpose — There are reports on perioperative deaths in cemented total hip arthroplasty (THA), and THA revisions are associated with increased mortality. We compared perioperative (intraoperatively or within 3 days of surgery), short-term and long-term mortality after all-cemented, all-uncemented, reverse hybrid (cemented cup and uncemented stem), and hybrid (uncemented cup and cemented stem) THAs.

Patients and methods — We studied THA patients in the Norwegian Arthroplasty Register from 2005 to 2018, and performed Kaplan–Meier and Cox survival analyses with time of death as end-point. Mortality was calculated for all patients, and in 3 defined risk groups: high-risk patients (age ≥ 75 years and ASA > 2), intermediate-risk patients (age ≥ 75 years or ASA > 2), low-risk patients (age < 75 years and ASA ≤ 2). We also calculated mortality in patients with THA due to a hip fracture, and in patients with commonly used, contemporary, well-documented THAs. Adjustement was made for age, sex, ASA class, indication, and year of surgery.

Results — Among the 79,557 included primary THA patients, 11,693 (15%) died after 5.8 (0–14) years’ follow-up. Perioperative deaths were rare (30/10^5^) and found in all fixation groups. Perioperative mortality after THA was 4/10^5^ in low-risk patients, 34/10^5^ in intermediate-risk patients, and 190/10^5^ in high-risk patients. High-risk patients had 9 (CI 1.3–58) times adjusted risk of perioperative death compared with low-risk patients. All 4 modes of fixation had similar adjusted 3-day, 30-day, 90-day, 3–30 day, 30–90 day, 90-day–10-year, and 10-year mortality risk.

Interpretation — Perioperative, short-term, and long-term mortality after primary THA were similar, regardless of fixation type. Perioperative deaths were rare and associated with age and comorbidity, and not type of fixation.

Perioperative (intra- or early postoperative) deaths have been reported in cemented total hip arthroplasty (THA) and hemiarthroplasty of the hip (Sierra et al. 2009, Talsnes et al. 2013, Garland et al. 2017). One reason for early mortality may be the so-called Bone Cement Implantation Syndrome (BCIS) (Donaldson et al. 2009, Olsen et al. 2014). The symptoms of BCIS are hypoxia, with or without hypotension, and/or unexpected loss of consciousness occurring at or shortly after the time of cementation, mostly in old patients with some comorbidity, and may be fatal (Olsen et al. 2014).

Death is undisputedly an important adverse outcome. Thus, studying the superior mode of fixation in THA, one should also consider short-term and long-term mortality in addition to long-term revision rates. However, the complexity of several outcomes can make it difficult to conclude which fixation to choose for the individual patient scheduled for elective THA.

We compared perioperative-, short-term, and long-term mortality for patients after primary, all-cemented, all-uncemented, reverse hybrid (cemented cup and uncemented stem), and hybrid (uncemented cup and cemented stem) THAs using the Norwegian Arthroplasty Register (NAR).

## Patients and methods

Since its inception in 1987, the NAR has registered information on primary THAs and THA revisions in Norway. Among the data collected are: the patient’s identity, date of operation, indication for THA, type of implants, method of fixation, intraoperative complications, and other surgery-related factors. In addition, patient-related factors like age, sex, and comorbidity are collected. The NAR uses the unique identification number of each Norwegian to link the primary THA to any subsequent revisions, and to the National Population Register, which provides information on death or emigration. The surgeons fill in the register form immediately after surgery and mail it to the NAR, where the data are entered electronically (Havelin et al. 2000). The data are validated, with 97% completeness of reporting of primary THAs, 88% reporting of revisions, 100% coverage of Norwegian hospitals, and 100% reporting of deaths (Furnes et al. 2019).

**Figure 1.**
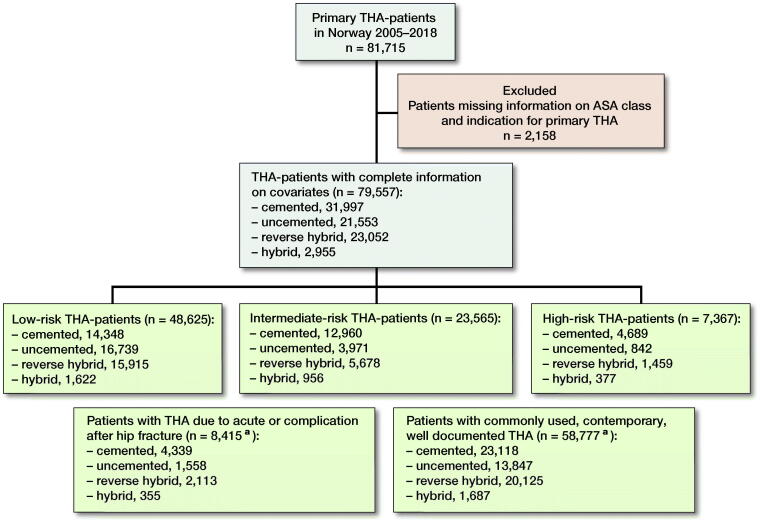
Flowchart of inclusion and exclusion of total hip arthroplasty (THA) patients. Patients in sub-groups are highlighted by green boxes. **^a^**Patients in these subgroups are also included in the 3 risk groups.

We assessed mortality after primary THAs registered with complete information on patient characteristics. The NAR has registered ASA class since 2005. Therefore, the period of inclusion and observation for the present study was from January 1, 2005 to December 31, 2018. Patients were included with their 1st primary THA only, if they had been subject to bilateral primary THA. From 2005 to 2018 the NAR contained data on 81,715 primary THA patients, 79,557 were eligible for analyses (Figure 1).

### Statistics

We compared patients with cemented, uncemented, reverse hybrid or hybrid THAs by Kaplan–Meier (KM) survival analyses. Furthermore, we performed adjusted survival analyses using Cox regression models, adjusted for age, sex, ASA class, and indication for primary THA. Additionally, we adjusted for the year of primary surgery to minimize the effect of time-dependent confounding.

All patients in each fixation group were followed until date of death or emigration, or until December 31, 2018. We ignored revisions in the analyses, and estimated adjusted hazard rate ratios, as a measure of relative risk, with 95% confidence intervals (CI), for modes of fixation and covariates. Primary outcomes were perioperative mortality (intraoperative death or death within 3 days of THA) and 10-year mortality. Secondary outcomes were 30-day, 90-day, 3–30-day, 30–90-day, 90-day–10-year, and 10-year mortality. 3 risk groups were assessed: low-risk patients (age < 75 years and ASA ≤ 2), intermediate-risk patients (age ≥ 75 years or ASA > 2), high-risk patients (age ≥ 75 years and ASA > 2). In addition, we assessed 2 subgroups independently of the risk groups: (1) patients with THA due to acute or complications after hip fracture, an emerging indication for THA; (2) patients who received commonly used, contemporary, and well-documented THAs, comparable to a separate study on implant survival, since mortality is being used as a reason for choice of fixation principle (Dale et al. 2019). These two latter subgroups were also included in the risk groups. Since perioperative deaths were few, comparisons of risk factors were also performed by Fisher’s exact test with and without Bonferroni multiple comparison correction.

**Figure 2.**
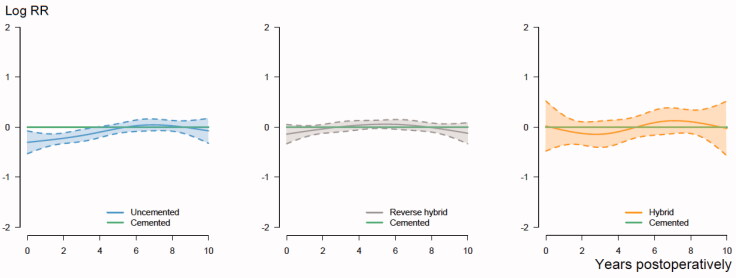
The relationship between year postoperatively and the log relative risk (RR) of death for uncemented, reverse hybrid and hybrid THAs, compared with cemented THAs, with 95% confidence intervals. The horizontal green line shows the reference relative risk (RR = 1) of cemented THAs. It is adjusted for age, sex, ASA class, indication for primary THA, and year of primary THA in the analyses. The course is proportional if lines are parallel.

We performed the analyses in concordance with the guidelines for statistical analyses of arthroplasty register data (Ranstam et al. 2011). The proportional hazard assumptions of the Cox survival analyses were largely fulfilled, when the smoothed Schoenfeld residuals were visually inspected (Figure 2). Competing risk models were not considered, since we ignored revision surgery. Bilaterality was not relevant since patients were included only with their 1st primary THA.

We considered non-overlapping 95% confidence intervals (CI) as statistically significant. The IBM SPSS 24.0 (IB Corp, Armonk, NY, USA) and R statistical software (R Centre for Statistical Computing, Vienna, Austria) packages were used for analyses. The study was performed in accordance with the RECORD and STROBE statement.

### Ethics, data sharing plan, funding, and potential conflicts of interests

The registration of data and the study were performed confidentially on patient consent and according to Norwegian and EU data protection rules. Data may be accessible upon application to the NAR. The study was fully financed by the NAR, and no conflict of interest is declared.

## Results

During the study period, 11,693 (15%) of the 79,557 primary THA patients died. In general, patients who had cemented THA were older and with more comorbidity than those who had uncemented THA. Patients receiving reverse hybrid or hybrid THAs were intermediate groups concerning age and comorbidity. Patients with uncemented, reverse hybrid, and hybrid THAs had shorter median follow-up due to increased use of these fixation modes towards the end of the study period (Tables 1 and 2).

**Table 1.** Demographic risk factors by type of fixation

Risk factors	Number of THA patients n = 79,557	Dead patients (%) n = 11,693 **^a^**	Cemented THA (%) n = 31,997	Uncemented THA (%) n = 21,553	Reverse hybrid THA (%) n = 23,052	Hybrid THA (%) n = 2,955
Sex						
Male	28,512	4,450 (16)	31	42	38	36
Female	51,052	7,243 (14)	69	58	62	64
Age						
< 45	2,843	57 (2)	1	8	3	2
45–54	6,874	209 (3)	2	17	10	7
55–64	18,876	1,122 (6)	14	32	30	22
65–74	28,235	3,277 (12)	39	30	36	33
75–84	19,156	5,283 (28)	37	11	17	30
≥ 85	3,573	1,745 (49)	7	1	3	6
ASA class						
1	16,063	1,281 (8)	15	27	21	15
2	47,899	5,761 (12)	59	59	62	63
3	15,247	4,465 (29)	25	17	17	21
4	348	195 (56)	1	0.3	0.3	1
Indication for primary THA						
Osteoarthritis	59,285	8,015 (14)	76	71	77	67
Inflammatory hip disease	1,722	269 (16)	2	2	2	2
Acute hip fracture	3,187	661 (21)	5	2	4	5
Complication after hip fracture	5,208	1,735 (33)	8	5	5	7
childhood hip disease	7,266	394 (5)	4	16	8	15
Osteonecrosis of the femoral head	2,268	442 (19)	3	3	3	3
Other diagnosis	621	177 (29)	1	1	1	1

a Dead overall 15%

**Table 2.** Main characteristics of the included THA patients by types of fixation

	All n = 79,557	Cemented n = 31,997	Uncemented n = 21,553	Reverse hybrid n = 23,052	Hybrid n = 2,955
Dead at 10 years, n (%)	10,330 (13)	6,843 (21)	1,260 (6)	2,018 (9)	209 (7)
Mean follow-up (range), years	5.8 (0–14)	6.9 (0–14)	4.9 (0–14)	5.5 (0–14)	3.8 (0–14)
Median follow-up (IQR), years	5.4 (2.6–8.8)	7.0 (3.4–10)	4.2 (1.8–7.6)	5.2 (2.9–7.7)	2.6 (1.1–4.9)
Mean age (range)	69 (11–97)	73 (22–98)	62 (11–95)	66 (14–97)	70 (17–97)
Mean ASA-class	2.0	2.1	1.9	2.0	2.1
At risk after 10 years	13,924	8,811	2,387	2,402	324

IQR = Inter quartile range

### Perioperative mortality

Perioperative deaths were rare (30/10^5^) and found in all modes of fixations. Adjusted 3-day mortality risk was similar after cemented, uncemented, reverse hybrid, and hybrid THAs (Table 3, see Supplementary data). The mortality on the day of surgery was 10/10^5^.

**Table 4.** Risk factors of perioperative death. Adjusted for age, sex, ASA class, indication for primary THA, and year of primary surgery

Risk factor	THAs	Deaths within 3 days	3-day relative mortality risk (CI)
Sex			
Female	51,045	18	1
Male	28,512	6	0.7 (0.3–1.9)
ASA class			
1	16,063	0	
2	47,899	8	1
3	15,247	15	3.2 (1.3–7.7)
4	348	1	5.2 (0.6–45)
Age group			
< 45 years	2,843	0	
45–54	6,874	0	
55–64	18,876	1	0.6 (0.1–5.6)
65–74	28,235	3	1
75–84	19,156	12	4.4 (1.2–16)
≥ 85	3,573	8	9.4 (2.4–37)
Indication for primary THA			
Osteoarthritis	59,285	10	1
Inflammatory hip disease	1,722	0	
Acute hip fractures	3,187	5	9.4 (3.1–28)
Complication after hip fracture	5,208	6	3.1 (1.1–8.7)
childhood hip disease	7,266	1	2.8 (0.4–22)
Osteonecrosis of the femoral head	2,268	1	2.4 (0.3–19)
Other diagnosis	621	1	7.2 (0.9–59)
Risk class^a^			
Low	48,625	2	1
Intermediate	23,565	8	2.7 (0.4–16)
High	7,353	14	8.7 (1.3–58)

a Adjusted for age, sex, indication for primary THA, and year of primary surgery.

Low = age < 75 years and ASA ≤ 2

Intermediate = age ≥ 75 years or ASA > 2

High = age ≥ 75 years and ASA > 2

THA patients with ASA 3 (few patients with ASA 4), age over 75 years, or THA in the course of a hip fracture had a higher risk of perioperative death after THA (Table 4).

When stratified into risk groups, perioperative mortality after THA was 4/10^5^ in low-risk patients, 34/10^5^ in intermediate-risk patients, and 190/10^5^ in high-risk patients. High-risk patients had nearly 9 times the risk of adjusted perioperative death after primary THA compared with low-risk patients (Table 4). We found no statistically significant difference in risk of perioperative death between the 4 modes of fixation, in either of the 3 risk groups or 2 subgroups (Table 3, see Supplementary data). That was also the finding when assessing perioperative death in the 4 fixation groups by Fisher’s exact test with Bonferroni multiple comparison correction. In Fisher’s exact test without correction, and not adjusted for patient characteristics, uncemented THA had lower perioperative mortality, compared with cemented (p = 0.03). The 24 patients who died were older and more comorbid (Table 5, see Supplementary data).

### Short- and long-term mortality

**Figure 3.**
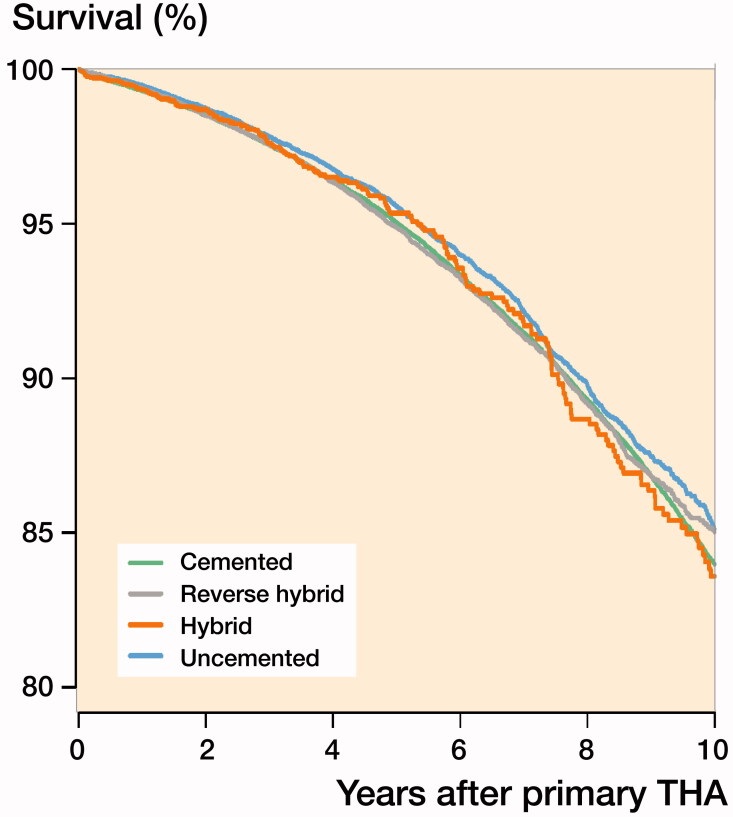
Patient survival curve for the 4 modes of fixation adjusted for age, sex, ASA class, indication for primary THA, and year of primary surgery.

The 10-year mortality risk and adjusted mortality was similar regardless of fixation (Table 3, Figure 3). In addition, we found similar results for all 4 fixation groups concerning 30-day, 90-day, 3–30-day, 30–90-day, 90-day–10-year, and 10-year mortality, and short- and long-term mortality did not change throughout the study period. This was also true for the finding in the “best-case” group of commonly used, contemporary, well-documented THAs.

## Discussion

Both the 3-day and the 10-year mortality were similar after all-cemented, all-uncemented, reverse hybrid, and hybrid THA. Risk factors for perioperative death were patient-related and not related to mode of fixation. This was also the finding when assessing high-risk patients only.

Traditionally, uncemented THAs have had lower implant survival than cemented THAs in register studies (Hailer et al. 2010, Mäkelä et al. 2014). This was also the case in the revision study based on most of the patients included in the present study (Dale et al. 2019). Nevertheless, the use of uncemented fixation in THA is increasing, even in old and frail patients (Troelsen et al. 2013, Mäkelä et al. 2014, Furnes et al. 2019). 1 of the explanations for this trend may be the fear of sudden perioperative deaths because of 3rd generation cementing technique (pressure cementation) and BCIS. This is a well-known complication associated with the use of bone cement, but the incidence is low in primary THA (Donaldson et al. 2009, Sierra et al. 2009). BCIS have also been highlighted in papers on cemented hemiarthroplasty used in old and frail femoral neck fracture patients (Talsnes et al. 2013, Olsen et al. 2014, Rutter et al. 2014).

Acknowledging the existence of BCIS, and that modern intensive care medicine can successfully treat the acute and potentially fatal BCIS, we considered deaths within 3 days postoperatively as potentially associated with the cementation, although other causes of death were also possible as a result of the surgical trauma.

Information on the cause of death was not available in our patients. Sierra et al. (2009) reported 3/10,000 perioperative deaths after cemented THA on the day of surgery, which was higher than our finding (1/10,000). Pripp et al. (2014) found that only half of the deaths on the day of cemented arthroplasty might be attributed to BCIS. A study from the Finnish Hospital Discharge register found similar 2-day mortality for patients after cemented, uncemented, and hybrid THA (Ekman et al. 2019). A Swedish study reported a small, but statistically significant increased relative risk of death in patients during the first 14 days after cemented THA, compared with uncemented THA (Garland et al. 2017). We did not find such a difference, even in the high-risk subgroup or in the group of patients with THA in the course of a hip fracture. However, we adjusted for comorbidity by ASA class, whereas Garland et al. adjusted by modified Charlson Comorbidity Index, which may have slightly different effect (Lavelle et al. 2015).

The potential risk of perioperative death needs to be weighed against other outcomes, such as the risk of revision due to fracture, dislocation, and infection. Revision risk due to these causes is higher after uncemented THAs (Hailer et al. 2010, Stea et al. 2014, Dale et al. 2019). Such complications may implicate a poorer functional outcome, increased morbidity, and long-term mortality (Lindahl et al. 2007, Gundtoft et al. 2017, Cnudde et al. 2019).

In the subgroup of commonly used, contemporary, well-documented THAs, the differences in risk of revision between the 4 modes of fixation, presented in a separate paper, were quite small (Dale et al. 2019). The exception was that uncemented THA in females over 55 years of age had a higher risk of revision due to aseptic loosening, periprosthetic fracture, and dislocation. Patients may have increased long-term mortality after such revisions (Gundtoft et al. 2017, Cnudde et al. 2019). 3-day and 10-year mortality was similar for all fixations in our study, indicating that perioperative-, short-term, or long-term mortality risk should not dictate what fixation principle to choose in primary THA, even in high-risk patients.

Mortality is low in healthy old patients elected for THA (Lie et al. 2000, Jamsen et al. 2013). We found a 10-year mortality of 15–16% for all modes of fixation, and 7–8% in the low-risk class. However, Lie et al. (2010) found an excess mortality of 0.12% in the first 26 days after primary THA compared with the baseline mortality for these patients. From 70 days and onwards THA patients have been reported to have lower mortality compared with the baseline population (Lie et al. 2002). Hunt et al. (2013) reported a yearly decreasing 90-days mortality after contemporary THA in the National Joint Registry for England and Wales between 2003 and 2011, and similar results concerning 90-days mortality after all 4 modes of fixation. The decreased mortality was attributed to improved perioperative prophylaxis and treatment. We did not find a similar decrease in mortality, but several of the modifiable factors suggested, such as chemical thromboembolic prophylaxis and spinal anesthesia, were used in the vast majority of THAs in Norway during the study period. Mortality from 30 days after primary THA, regardless of mode of fixation, has been found to be lower than for controls (matched population without THA) (Pedersen et al. 2011, Garland et al. 2017). Either patients chosen for elective THA are healthier than the average population, or one may say that successful THA may protect the patients and result in lower mortality than the corresponding population (Cnudde et al. 2018). Probably very frail patients are not selected for elective THA.

### Strengths and limitations

Our study is based on validated data from the NAR, with high completeness of reporting of primary THAs and deaths (Furnes et al. 2019). We therefore had the benefit of complete information on patient-related risk factors. Accordingly, we were able to adjust for important differences between the patient groups. Risk factors we included in the analyses such as age, ASA class, and indication for primary THA are associated with adverse outcomes and mortality after surgery (Belmont et al. 2014, Cnudde et al. 2018). The effect of adjustment for such risk factors on 10-year KM survival after cemented THA illustrates the importance including age and comorbidities in assessments of mortality.

Perioperative deaths are rare, and it may only be possible to study these in large databases such as arthroplasty registers. We included a large number of THAs with exact survival times of the patients. Since results are from a nationwide THA population, external validity should be good.

Register studies have inherent limitations (Varnum et al. 2019). A limitation in our study was the limited information on the cause of perioperative death. We therefore considered deaths within 3 days postoperatively as potentially associated with the cementation. This was an approximation, and Pripp et al. (2014) found that only half of perioperative mortalities are attributed to BCIS. This indicates that the number of perioperative deaths caused by BCIS in our study would be even lower for THAs involving bone cement. In Finland, where uncemented THA is more common, even in old and frail patients, similar perioperative mortality has been reported after cemented and uncemented THAs (Ekman et al. 2019).

The follow-up is relatively short and different for the 4 fixation groups, since there has been a shift towards more use of uncemented, reverse hybrid and hybrid THAs (see Table 2) (Furnes et al. 2019). We did not find differences in perioperative, short-, or long-term mortality. Limited follow-up should therefore have a small effect, if any, on the results.

### Conclusion

Results after contemporary primary THA may be good regardless of fixation, but uncemented THA had an increased risk of revision in a cohort of patients also included in the present study (Dale et al. 2019). Perioperative deaths, however, were associated only with patient-related risk factors, like age and comorbidity, and not surgery-related factors like fixation. Sudden death will be the most serious adverse event, but death during a course of complications is equally serious. We found that perioperative, short-term, and long-term mortality after primary THA were similar, regardless of fixation mode. Use of bone cement appears to be safe in all patient groups. Perioperative deaths were associated with advanced age and comorbidity, and not type of fixation, and should therefore not guide the choice of fixation in primary THA, even in high-risk patients.

### Supplementary data

Tables 3 and 5 are available as supplementary data in the online version of this article, http://dx.doi.org/10.1080/17453674. 2019.1701312

## Supplementary Material

Supplemental Material
