# Supplementary material for: Perioperative, short-, and long-term mortality related to fixation in primary total hip arthroplasty: a study on 79,557 patients in the ­Norwegian Arthroplasty Register
Source: Acta Orthop. 2019 Dec 13;91(2):152–8. doi: 10.1080/17453674.2019.1701312 (PMC7155214; doi:10.1080/17453674.2019.1701312)
Supplement: Supplemental Material [file IORT_A_1701312_SM0881.pdf]

Supplementary data

Table 3. Overall 3-day and 10-year mortality risk for the 4 groups of fixation, adjusted for age, sex, ASA class, indication for primary THA, and year of primary surgery, in addition to estimated 10-year Kaplan–Meier (K–M) and adjusted Cox mortality

	THAs	Deaths within 3 days	3-day relative mortality risk (CI)	Deaths within 10 years	10-year relative mortality risk (CI)	10-year K–M mortality (%)	Adjusted 10-year mortality (%)	At risk after 10 years
All patients								
Cemented	31,997	16	1	6,843	1	32 (32–31)	16 (15–17)	8,811
Uncemented	21,553	3	1.0 (0.3–3.4)	1,260	0.9 (0.9–1.0)	14 (14–15)	15 (14–16)	2,387
Reverse hybrid	23,052	4	0.7 (0.2–2.3)	2,018	1.0 (0.9–1.0)	17 (16–18)	15 (14–16)	2,401
Hybrid	2,955	1	0.8 (0.1–5.7)	209	1.0 (0.9–1.1)	22 (19–15)	16 (14–19)	324
Low risk patients								
Cemented	14,347	1	1	1,389	1	14 (13–15)	8 (7–9)	5,364
Uncemented	16,739	1	1.9 (0.1–40)	477	0.9 (0.8–1.0)	7 (7–8)	7 (6–8)	2,022
Reverse hybrid	15,915	0	–	648	0.9 (0.8–1.0)	8 (8–9)	7 (6–8)	2,073
Hybrid	1,622	0	–	52	1.0 (0.7–1.3)	10 (7–12)	8 (6–10)	251
Intermediate risk patients								
Cemented	12,960	7	1	3,416	1	41 (40–42)	34 (32–35)	2,916
Uncemented	3,971	1	1.0 (0.1–8.7)	526	0.9 (0.8–1.0)	33 (30–36)	32 (29–35)	340
Reverse hybrid	5,678	0	–	886	1.0 (0.9–1.1)	34 (32–37)	32 (29–34)	303
Hybrid	956	0	–	90	1.0 (0.8–1.3)	38 (30–46)	35 (27–42)	65
High risk patients								
Cemented	4,689	8	1	2,038	1	67 (65–68)	67 (64–69)	531
Uncemented	842	1	1.2 (0.2–10)	257	1.0 (0.9–1.2)	67 (60–74)	69 (61–76)	25
Reverse hybrid	1,459	4	3.3 (0.9–12)	484	1.1 (1.0–1.2)	67 (61–72)	68 (62–74)	26
Hybrid	377	1	3.6 (0.4–31)	67	1.0 (0.7–1.2)	70 (57–83)	65 (52–78)	8
Patients with THA due to acute or complications after hip fractures								
Cemented	4,339	8	1	1,525	1	54 (52–56)	39 (36–42)	711
Uncemented	1,588	2	2.4 (0.5–12)	210	0.9 (0.8–1.0)	29 (26–33)	37 (32–43)	177
Reverse hybrid	2,113	1	0.5 (0.1–4.5)	437	1.0 (0.9–1.1)	35 (32–39)	35 (31–39)	179
Hybrid	355	0	–	64	1.2 (1.0–1.6)	50 (39–61)	44 (33–66)	22
Patients with contemporary well-documented THAs [a]								
Cemented	23,118	9	1	4,634	1	30 (29–31)	14 (14–15)	5,400
Uncemented	13,847	0	–	875	1.0 (0.9–1.2)	15 (14–16)	15 (14–17)	1,596
Reverse hybrid	20,125	1	0.5 (0.1–4.5)	1,844	1.1 (1.0–1.1)	17 (16–18)	14 (13–15)	1,656
Hybrid	1,687	1	2.4 (0.2–32)	77	1.0 (0.8–1.2)	12 (9–16)	15 (13–20)	138

[a] Adjusted for surgical approach, articulation, and head size of the prosthesis in addition.

Table 5. Patient and procedure characteristics of THA-patients who died perioperatively (intraoperatively or within 3 days of surgery)

Dead on day	Sex	Age	ASA class	Indication for primary THA	Duration of surgery (min)	Fixation	Peroperative complications	Risk class
0	Female	84	3	Complication after hip fracture	255	Cemented		High
0	Female	72	2	Osteonecrosis of the femoral head	116	Cemented		Low
0	Female	81	2	Osteoarthritis	120	Cemented		Intermediate
0	Female	82	4	Acute hip fracture	95	Cemented	Pulmonary embolus	Intermediate
0	Male	88	3	Osteoarthritis	100	Reverse hybrid		High
0	Female	93	2	Acute hip fracture	160	Cemented		Intermediate
0	Female	81	3	Complication after childhood hip disease	90	Hybrid	Cerebral stroke	Intermediate
0	Female	67	2	Osteoarthritis	60	Uncemented		Low
1	Female	66	3	Acute hip fracture	146	Cemented		Intermediate
1	Female	84	3	Osteoarthritis	105	Cemented		High
1	Female	85	2	Complication after hip fracture	120	Cemented		Intermediate
1	Male	78	3	Osteoarthritis	70	Cemented		Intermediate
1	Female	88	3	Acute hip fracture	61	Uncemented		High
1	Female	88	2	Complication after hip fracture	107	Cemented	Cement reaction	Intermediate
1	Female	64	3	Complication after hip fracture	150	Uncemented		Intermediate
1	Female	87	3	Other but unknown	225	Reverse hybrid		High
1	Male	90	2	Osteoarthritis	314	Cemented	Excessive bleeding	Intermediate
2	Female	76	2	Osteoarthritis	85	Cemented		Intermediate
2	Male	85	3	Osteoarthritis	90	Cemented		Intermediate
2	Female	84	3	Osteoarthritis	58	Cemented		High
2	Female	88	3	Osteoarthritis	90	Reverse hybrid		High
2	Male	75	3	Complication after hip fracture	90	Cemented		High
2	Female	83	3	Complication after hip fracture	180	Reverse hybrid		High
2	Male	80	3	Acute hip fracture	95	Cemented		High
